# Overexpression of Citron Rho-Interacting Serine/Threonine Kinase Associated with Poor Outcome in Bladder Cancer

**DOI:** 10.7150/jca.43435

**Published:** 2020-04-14

**Authors:** Jiafeng Shou, Chong Yu, Dahong Zhang, Qi Zhang

**Affiliations:** Department of Urology, People's Hospital of Hangzhou Medical College, Zhejiang Provincial People's Hospital, 158 Shangtang Road, Hangzhou, Zhejiang Province 310014, People's Republic of China

**Keywords:** bladder cancer, CIT, prognosis, survival, biomarker

## Abstract

**Objective:** Citron Rho-Interacting Serine/Threonine Kinase (CIT) was originally identified as a binding partner of active forms of the small GTPases Rho and Rac. This kinase participated in the regulation of cytokinesis and loss of CIT was associated with chromosomal instability. Here, we assume that CIT might be a potential prognostic biomarker for bladder cancer.

**Materials and Methods:** The expression and prognostic significance of CIT mRNA were validated on 5 published microarray data sets, including 948 bladder cancer cases. To further confirm the results, we collected 54 non-carcinomatous human bladder tissue samples and 315 bladder cancer tissues from Zhejiang Provincial People's Hospital to detect the protein level of CIT based on the immunohistochemistry analysis. The Kaplan-Meier method and Cox proportional hazards regression model were used in survival analysis.

**Results:** Analysis results showed that high CIT expression was associated with tumor size (p=0.0001), tumor grade (p<0.0001), smoking status (p=0.0143), TNM stage (p=0.0024), pathological tumor stage (p<0.0001) and aggressive phenotypes of bladder cancer. Independent and pooled survival analyses both indicated that overexpression of CIT was significantly associated with poor survival of bladder cancers.

**Conclusions:** In conclusion, these findings indicated that overexpression of CIT was significantly associated with poor survival outcome in bladder cancers. CIT might serve as a promising prognostic biomarker and therapeutic target for bladder cancers.

## Introduction

Citron Rho-Interacting Serine/Threonine Kinase (CIT), originally identified as a RhoA effector that could regulate myosin contractility by phosphorylating the myosin regulatory light chain, is localized at the cleavage furrow and at the midbody of dividing cells^1^,^2^. CIT binds to Rho-GTP and have been shown to be involved in the regulation of cytokinesis 3-5. Loss of CIT causes failure of cytokinesis and therefore triggers apoptosis in the male germ cells and a specific population of neuroblasts 6,7. In addition, CIT is demonstrated as a cell cycle dependent, nuclear protein required for G2/M transition of hepatocytes 8. Predictably, imbalance of cell cycle is commonly selected for in evolving cancer cells 9. Thus, it would be of significance to investigate the clinical role of CIT for cancer control.

Bladder cancer is a common urinary malignancy worldwide. In the United States, Bladder cancer is expected to take up 7% of all new cancer cases and 4% of all cancer deaths in men 10. According to cancer statistics of the United States, bladder cancer is estimated to be the second most frequent genitourinary tract cancer and the fourth most common cancer in male in 2017 10. Bladder cancer is generally categorized into two groups: superficial bladder cancer and muscle-invasive bladder cancer (MIBC). Despite radical cystectomy and neoadjuvant chemotherapy applied in bladder cancer, the prognosis is still poor due to its recurrent nature 11. New and more effective therapeutic strategies are urgently needed for bladder cancer. Here, we hypothesize that CIT could serve as prognostic biomarker and therapeutic target in bladder cancer treatment.

## Materials and Methods

All methods were carried out in accordance with relevant guidelines and regulations which are in compliance with institutional, national, or international guidelines.

### Differential expression and coexpression of CIT in bladder cancer

To identify differentially expressed genes in bladder cancers, we analyzed the microarray data set available in the Oncomine database. (www.oncomine. org; accessed on September 30, 2017). The key words used were Gene: “CIT”, Cancer Type: “bladder cancer”, Analysis Type: “Cancer vs. cancer Analysis” and “Coexpression Analysis”. Detailed information about tissue collection and the experimental protocol of each study is available in the Oncomine database or from the original publications.

#### Associations of CIT expression with clinical characteristics and prognosis of patients with bladder cancer

Microarray data sets: A total of 5 published microarray data sets containing survival information of bladder cancer patients was downloaded from the Array Express database (www.ebi.ac.uk/arrayexpress) including GSE13507, GSE31684, E-MTAB-1803 and E-MTAB-4321, and TCGA-BLCA was downloaded from The Cancer Genome Atlas (TCGA)(www.cancergenome.nih.gov). These data sets were used to further evaluate the role of CIT in bladder cancer progression and prognosis. Detailed information of the microarray data sets is summarized in supplementary Table 1 (Table S1). The overall survival (OS) was calculated as the time from initial surgery to the date of death from any cause. The cancer-specific survival (CSS) was calculated as the time from initial surgery to the date the patient was last seen, and only deaths from bladder cancer were considered as the end of the survival period. The progression-free survival (PFS) was defined as the time from initial surgery until tumor progression to T2+. The recurrence-free survival (RFS) was defined as the time from initial surgery until tumor recurrence. To normalize the mRNA expression levels among the included data sets, we re-stratified the scores of CIT and other related genes into four grades (Q1, Q2, Q3 and Q4) based on the percentile for each independently downloaded data set. Subgroup of Q1 was 0 to 25% percentile; Q2 was 25% to the median; Q3 was the median to 75% percentile; and Q4 was 75% percentile to maximum. For further analysis, less than the value of the median was regarded as CIT-low, and greater or equal to the median was CIT-high 12.

### Gene set enrichment analysis

To evaluate the correlations between CIT expression and cancer-related pathways, gene set enrichment analysis (GSEA) was performed using the above bladder cancer microarray data set GSE31684. The detailed protocol of GSEA was available on the Broad Institute Gene Set Enrichment Analysis website (www.broad.mit.edu/gsea). Briefly, data sets and phenotype label files were created and loaded into GSEA software (v2.0.13). The gene sets were downloaded from the Gene Expression Omnibus GEO (http://www.cancergenome.nih.gov/geo/). Gene set permutations were performed 1000 times for each analysis. The normalized enrichment score (NES) was calculated for each gene set. GSEA results with a nominal P < 0.05 were considered significant 13.

### Cell lines and culture conditions

The human bladder cancer cells T24 (Grade III) and RT4 (Grade I) cell lines were purchased from American Tissue Culture Collection. T24 cells were cultured in MEM media and RT4 cells were cultured in McCoy's media. All media were supplemented with 10% FBS (Atlanta Biologicals Flowery Branch, GA, USA), 100 I.U. penicillin, and 100 µg/ml streptomycin and grown in an atmosphere of 5%CO_2_ at 37°C.

### Western blotting assays

Total protein was extracted from cells lysed with the M-PER Mammalian Protein Extraction Reagent (Thermo) supplemented with the cocktail of protease inhibitors (Sigma). After blocking with 5% non-fat milk in Tris-buffered saline with Tween-20 (TBST) for 60 min, the membrane was incubated with the primary antibodies anti-human-CIT (1:2000, Abcam, UK) or anti-human ß-actin (1:5000, Santa Cruz Biotechnology, USA) dissolved in 5% bovine serum albumin in TBST overnight at 4°C.

### Quantitative immunohistochemistry assays

Immunohistochemical analysis was performed to study CIT protein expression in 54 non-carcinomatous human bladder tissue samples and 315 human urothelial carcinoma tissues from Zhejiang Provincial People's Hospital. Our study was approved by the Human Research Ethics Committee of Zhejiang Provincial People's Hospital. Immunohistochemical (IHC) analysis was described in our previous study 14. In brief, slides were baked at 60℃ for 2 hours followed by deparaffinization with xylene and rehydrated. The sections were submerged in EDTA antigenic retrieval buffer and microwaved for antigen retrieval, after which they were treated with 3% hydrogen peroxide in methanol to quench endogenous peroxidase activity, followed by incubation with 1% bovine serum albumin to block nonspecific binding 14. The sections were then incubated with rabbit polyclonal anti-CIT (1:200; Abcam, Cambridge, MA, USA) at 4℃ for 16 hours. Secondary antibody was applied with the use of Envision (Rabbit, Dako, Denmark). Normal goat serum was used as negative control. After washing, tissue sections were treated with secondary antibody. Tissue sections were then counterstained with hematoxylin, dehydrated, and mounted. The degree of immune staining was reviewed and scored independently by 2 pathologists based on the intensity of staining who were unaware of the clinical and pathologic data. In cases of discrepancy, a consensus score was chosen for evaluation. An immunoreactive score (IRS) was calculated as described before 14. Staining intensity was graded according to the following criteria: 0 (no staining), 1 (weak staining=light yellow), 2 (moderate staining= yellowish brown), and 3 (strong staining=brown). Moderate and strong staining were used to define tumors with high CIT expressions, and no and weak staining were used to indicate low CIT expressions. Staining percentage was graded according to the proportion of positively staining tumor cells as follows: 0 for <5% positive tumor cells; 1 for 6% to 25% positive tumor cells; 2 for 26% to 50% positive tumor cells; and 3 for >51% positive tumor cells. We use this method of assessment to evaluate CIT expression in human non-tumor mucosa and malignant lesions by determining the staining index with scores of 0, 1, 2, or 3 14.

### Statistical analyses

All statistical analyses were performed using the SAS statistical software, version 9.2 (SAS Institution Inc., Cary, NC), unless otherwise noted. Student t-test and one-way ANOVA were used for continuous data analyses, and Pearson Chi-square test was used for categorical data analyses. Survival curves were generated using Kaplan-Meier methods, and the log-rank test was carried out to evaluate the survival differences between groups. Hazard ratios (HR) with 95% confidence intervals (CI) were calculated using Cox proportional hazards regression analysis to examine the association of CIT expression levels with patient survival. P<0.05 was considered statistically significant, and all P values were 2-sided.

## Results

### CIT expression is positively correlates with aggressive phenotypes of bladder cancer

**Figure 1:**
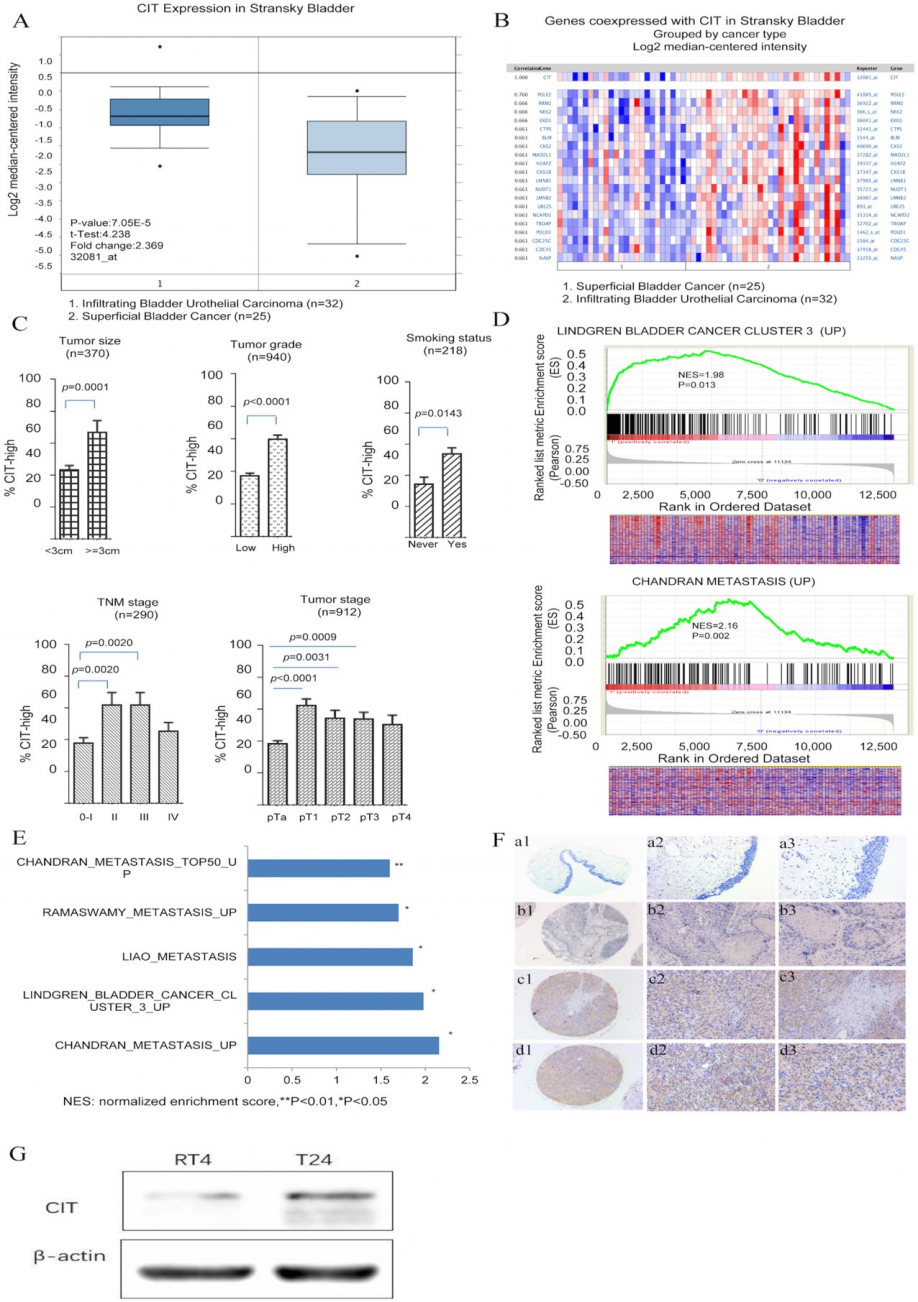
CIT is associated with poor differentiation and aggressive phenotypes of bladder cancer.** (A)** CIT mRNA expression in bladder cancer tissues from the Oncomine database. Stransky Bladder (n=57) was selected to analysis CIT mRNA expression in bladder tissues from infiltrating bladder urothelial carcinoma patients and superficial bladder cancer. The median-centered intensity log2 expression values of CIT were -1.192 in infiltrating bladder urothelial carcinoma (n=32) , -2.166 in superficial bladder cancer (n=25)(p<0.001). **(B)** A co-expression gene analysis was also performed in Stransky Bladder, the correlation with CIT was cut off at 0.661. Colors are z-score normalized to depict relative values within rows. Blue indicates least expressed, red indicates most expressed and grey indicates not measured. They cannot be used to compare values between rows. **(C)** High expression of CIT related to bladder cancer TNM stage, histological grades, tumor size and smoking status using downloaded published data sets. **(D)** Enriched gene signatures associated with aggressiveness and prognosis in CIT-high and CIT-low bladder cancers. NES represents the normalized enrichment score for the gene-set enrichment analyses (GSEA). **(E)** Selected gene sets that were enriched in the CIT-high tumors based on GSEA. **(F)** IHC staining for CIT in non-cancerous and cancerous bladder tissue. a1-a3: strong staining in non-cancerous bladder tissue. b1-b3: negative staining in urinary bladder carcinoma. c1-c3: strong staining in non-muscle-invasive urinary bladder carcinoma. d1-d3: strong staining in muscle-invasive urinary bladder carcinoma. Magnification: the original magnification ×40 (a1-d1), ×100 (a2-d2), and ×200 (a3-d3). (G) Increased CIT level was observed in T24 (Grade III) compared with RT4 (Grade I).

To explore the molecular mechanism of bladder carcinogenesis, we analyzed the global gene expression profile of a cohort of human bladder cancer tissues from the Oncomine database (Human Genome U95A-Av2 Array). Among the changed genes, a poorly defined gene, CIT was significantly unregulated in infiltrating bladder urothelial carcinoma (IBC) comparing with superficial bladder cancer (SBC) (P=7.05e-15) (Figure 1A and B). Furthermore, we analyzed the RNA-seq data from the Array Express database and TCGA database. Five datasets including GSE13507, GSE31684, E-MTAB-1803, E-MTAB-4321 and TCGA-BLCA, were selected to research on the associations of CIT expression with clinical outcome of patients with bladder cancer. Analysis results showed that high CIT expression was associated with tumor size (p=0.0001), tumor grade (p<0.0001) and smoking status (p=0.0143), TNM stage (p=0.0024) and pathological tumor stage (p<0.0001) (Figure 1C).

A further GSEA analysis indicated that gene signatures related to metastasis were enriched in CIT- high bladder cancer in the GSE31684 data set (Figure 1D and 1E). The normalized enrichment score (NES) were 1.98 and 2.16, respectively (P < 0.05). The correlation between CIT and aggressive phenotypes of bladder cancer were further validated. We also measured CIT expression in bladder cancer cell lines according to different invasiveness. As shown in Figure 1G, significantly increased CIT level was observed in T24 (Grade III) compared with RT4 (Grade I).

To further confirm the results of GSEA, immunohistochemical analysis was performed to study CIT protein expression in 54 non-carcinomatous human bladder tissue samples and 315 human urothelial carcinoma tissues from Zhejiang Provincial People's Hospital. Figure 1F showed representative IHC results of CIT. CIT was mostly expressed in the bladder cancer cell cytoplasm. As shown in Table 1, high CIT expression was associated with tumor size (p=0.002), number of tumor focus (p=0.001), invasion depth (p=0.007), lymph node status (p<0.001), distant metastasis (p=0.002) and histological grade (p<0.001).

### CIT was associated with poor survival outcome of bladder cancer patients

**Figure 2:**
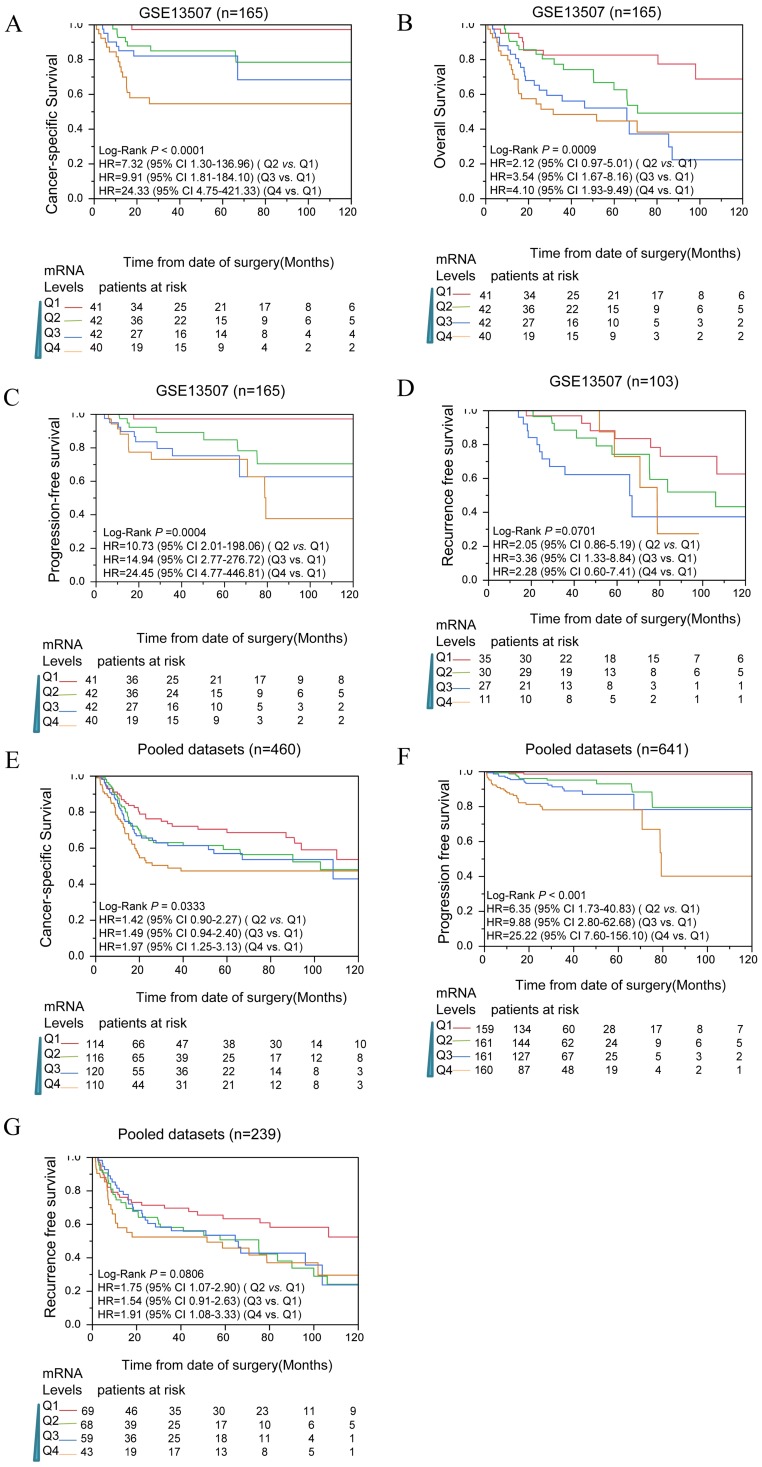
Kaplan-Meier analysis for CIT and outcome of bladder cancers among downloaded published data sets. Analysis results indicated that CIT impact the CSS**(A)**, OS**(B)**, PFS**(C)** and RFS**(D)** in a dose dependent manner in data sets. CSS**(E)**, PFS**(F)** and RFS**(G)** for CIT in all pooled data sets. In Kaplan-Meier plot figure, the curves of red, green, blue, and brown represented Q1, Q2, Q3 and Q4 subgroups respectively.

To determine whether CIT expression correlated with prognosis of bladder cancer patients, survival analyses were performed in public datasets. The expression levels of CIT were divided into four subgroups (Q1, Q2, Q3 and Q4). In Figure 2A, B, C and D, the mRNA levels of CIT significantly impacted poor CSS, OS, PFS and RFS of bladder cancer, respectively. Pooled analysis confirmed CIT has a significant impact on poor CSS, PFS and RFS of bladder cancer in all collected gene expression data sets. (Figure 2E, F and G).

Table 2 showed the survival analysis result that was conducted for each of data sets by using uni- and multiple Cox proportional hazard analysis. Q1, the lowest expression subgroup, was set as the relative point of reference. Mostly, it revealed that the HR of CIT for OS increased as CIT expression levels increased. Similar phenomenon was observed for PFS and RFS. The pooled analysis revealed that the HRs of Q4 for OS, PFS and RFS were 1.97 [95% confidence interval (CI): 1.25-23.13], 25.22 (95% CI: 7.60-156.10) and 1.91 (95% CI: 1.08-3.33), respectively.

The prognostic significance of CIT was also evaluated among patients from Zhejiang Provincial People's Hospital. The results were displayed in Table 3. In multivariate Cox proportional hazard analysis, the HR of CIT for OS was 3.115 (95% CI: 1.638-5.921, P = 0.001). It was confirmed that CIT expression acts as an independent factor for prediction of poor OS in bladder cancer patients. As for other factors, lymph node involvement (HR: 2.159, 95% CI: 1.459-3.196) and depth of invasion (T_2_-T_4_ vs Ta-T_1_) (HR: 1.472, 95% CI: 1.079-2.008) showed a significant worse outcome in OS.

## Discussion

To our knowledge, this is the first systemic research to analyze the correlation between CIT expression and prognosis in bladder cancers. In this study, we provided that higher mRNA and protein levels of CIT were significantly associated with aggressive phenotypes of bladder cancers and poorer prognosis. These findings indicate that CIT might be a driver gene in bladder cancers.

CIT kinase was originally identified as a binding partner of active forms of the small GTPases Rho and Rac 3. Subsequently, it was reported that CIT promote the constriction of the actomyosin contractile ring that drives cleavage furrow ingression during cytokinesis by phosphorylating the myosin regulatory light chain 1,15. Nevertheless, further studies revealed that CIT also played a role in later stages of cytokinesis after completion of furrow ingression 16-22. In the absence of CIT, the highly ordered arrangement of midbody proteins and the connection between the cortex and the central spindle microtubules are lost, which in turn leads to abscission failure 22. Mutation or loss of CIT had been proved to lead to defective neurogenesis in both mice humans 6,7,23-25. CIT loss can also cause chromosomal instability owing to its involvement in DNA damage control and involvement in cytokinesis 26. Chromosomal instability was a hallmark of many cancers that was associated with cancer evolution, diversification and heterogeneity, drug resistance and metastases 27-29. In addition, several studies had revealed that CIT depletion led to failure of cytokinesis and dramatically inhibited cell proliferation of liver, breast, cervical and colorectal cancer cell lines 30,31. This proliferation inhibition directly resulted from an increase in apoptosis via both p53- dependent and -independent pathways 31. Furthermore, dividing and polyploid cancer cells are more susceptible to CIT-K depletion 22,27. Therefore, CIT might be a promising therapeutic target for cancer treatment.

Despite valuable findings above, some limitations should be noted. First, we only showed the phenomenological impact of CIT on the prognosis of bladder cancers. The biological mechanisms between CIT and bladder cancer cells are warranted to be investigated in further studies. Second, we couldn't obtain direct information on socioeconomic factors, such as quality of medical care, socio-economic class, patients' education levels and compliance, which might significantly affect patient survival and outcome.

## Conclusion

In conclusion, these findings indicated that overexpression of CIT was significantly related to the poor survival outcome in bladder cancers. CIT could be a promising prognostic biomarker and therapeutic target for bladder cancers.

## Supplementary Material

Supplementary table S1.

## Figures and Tables

**Table 1:** Relationship of CIT expression with pathological parameters of bladder cancer

Clinical parameters	CIT			
	low	high	t/χ2	*P*
**Gender**			0.062	0.804
Male	139(87.4%)	262(88.2%)		
Female	20(12.6%)	35(11.8%)		
**Age (years)**			1.095	0.295
<60	60(34.7%)	112(39.6%)		
≥60	113(65.3%)	171(60.4%)		
**Size**			9.849	0.002
<3cm	102(59.0%)	124(43.8%)		
≥3cm	71(41.0%)	159(56.2%)		
**Number of tumor**			10.333	0.001
Single	50(28.9%)	46(16.3%)		
Multiple	123(71.1%)	237(83.7%)		
**Invasion depth**			7.292	0.007
Ta-T1	34(19.7%)	30(10.6%)		
T2-T4	139(80.3%)	253(89.4%)		
**Lymph node metastasis**			23.105	0.000
No	151(87.3%)	190(67.1%)		
Yes	22(12.7%)	93(32.9%)		
**Distant metastasis**			9.601	0.002
No	168(97.1%)	252(89.0%)		
Yes	5(2.9%)	31(11.0%)		
**Lymphovascular invasion**			1.599	0.206
Negative	149(93.7%)	268(90.2%)		
Positive	10(6.3%)	29(9.8%)		
**Histological grade**			17.537	0.000
Low grade	73(42.2%)	86(30.4%)		
High grade	100(57.8%)	197(69.6%)		

**Table 2:** Uni- and multivariate analysis for CIT and survival in microarray data sets

Data set		Overall survival		Progress-free survival		Recurrence-free survival	
		HR(95%CI)	Adjusted HR (95%CI)*	HR(95%CI)	Adjusted HR (95%CI)*	HR(95%CI)	Adjusted HR (95%CI)*
GSE13507							
	Q_1_	Reference	Reference	Reference	Reference	Reference	Reference
	Q_2_	7.32(1.30-136.96) †	5.29(0.92-99.83)	10.73(2.00-198.06) ‡	8.79(1.61-163.47) ‡	2.05(0.86-5.19)	1.86(0.75-4.87)
	Q_3_	9.91(1.81-184.10) ‡	4.32(0.70-83.40)	14.95(2.77-276.72) ‡	7.36(1.21-141.66) †	3.36(1.33-8.84) †	2.83(1.01-7.93) †
	Q_4_	23.33(23.33-421.33) ‡	8.52(1.46-162.52) †	24.45(4.77-446.81) ‡	9.89(1.63-191.09) ‡	2.28(0.60-7.41)	1.71(0.37-6.72)
GSE31684							
	Q_1_	Reference	Reference	N/A	N/A	Reference	Reference
	Q_2_	2.19(0.90-5.63)	2.38(0.97-6.14)			2.28(0.93-5.86)	2.69(1.08-7.05)
	Q_3_	1.29(0.49-3.47)	1.13(0.43-3.06)			1.31(0.53-3.39)	1.17(0.47-3.34)
	Q_4_	1.63(0.62-4.37)	1.40(0.53-3.78)			1.56(0.61-4.09)	1.31(0.51-3.46)
MTAB-4321							
	Q_1_	N/A	N/A	Reference	Reference	N/A	N/A
	Q_2_			2.90(0.37-58.67)	2.66(0.34-53.78)		
	Q_3_			6.94(1.24-129.79) †	6.12(1.09-114.55) †		
	Q_4_			24.73(5.15-443.60) ‡	16.27(3.25-295.98) ‡		
MTAB-1803							
	Q_1_	Reference	Reference	N/A	N/A	N/A	N/A
	Q_2_	1.00(0.40-2.42)	1.02(0.40-2.55)				
	Q_3_	1.59(0.68-3.79)	1.61(0.68-3.89)				
	Q_4_	1.55(0.63-3.80)	1.71(0.67-4.39)				
TCGA							
	Q_1_	Reference	Reference	N/A	N/A	Reference	Reference
	Q_2_	0.73(0.33-1.65)	0.59(0.26-1.36)			0.80(0.34-1.91)	0.74(0.31-1.81)
	Q_3_	0.87(0.38-2.02)	0.73(0.31-1.72)			1.62(0.56-4.56)	1.63(0.48-5.25)
	Q_4_	0.75(0.32-1.77)	0.65(0.27-1.56)			1.49(0.57-3.91)	1.18(0.39-3.53)
Pool set							
	Q_1_	Reference	Reference	Reference	Reference	Reference	Reference
	Q_2_	1.42(0.90-2.27)	1.28(0.81-2.04)	6.35(1.73-40.83) ‡	5.68(1.54-36.55) ‡	1.75(1.07-2.90) †	1.82(1.10-3.04) †
	Q_3_	1.49(0.94-2.40)	1.05(0.66-1.70)	9.88(2.80-62.68) ‡	7.40(2.07-47.17) ‡	1.54(0.91-2.63)	1.06(0.62-1.83)
	Q_4_	1.97(1.25-3.13) ‡	1.27(0.81-2.04)	25.22(7.60-156.10) ‡	15.01(4.35-94.59) ‡	1.91(1.08-3.33) †	1.10(0.61-1.95)

Note: Uni- and multivariate analysis were conducted to evaluate HR of CIT.* For multivariate analysis, HR was adjusted by Age and Grade. † Statistical significance, *P*<0.05; ‡ Statistical significance, *P*<0.01.

**Table 3:** Multivariate analysis of the correlation between clinicopathological parameters and survival time of patients with bladder cancer from Zhejiang Provincial People's Hospital

Covariates	Coefficient	Standard error	Hazard ratio (HR)	95.0% CI for HR	P
Age range (>60 vs ≤60)	0.464	0.437	1.590	0.676-3.741	0.288
Tumor size (≥3 cm vs <3 cm)	-0.071	0.151	0.932	0.692-1.253	0.639
Number of tumor (single vs multiple)	0.078	0.189	1.081	0.747-1.565	0.678
Lymph node metastasis (positive vs negative)	0.770	0.200	2.159	1.459-3.196	0.000
Lymphovascular invasion (positive vs negative)	0.042	0.271	1.043	0.613-1.773	0.877
Distant metastasis (positive vs negative)	0.384	0.274	1.469	0.859-2.511	0.160
CIT expression (high vs low)	1.136	0.328	3.115	1.638-5.921	0.001
Depth of invasion ( T_2_-T_4_ vs Ta-T_1_)	0.387	0.158	1.472	1.079-2.008	0.015
Histological grade	-0.052	0.130	0.949	0.736-1.224	0.688

## References

[1] Madaule P, Eda M, Watanabe N (1998). Role of citron kinase as a target of the small GTPase Rho in cytokinesis. Nature.

[2] Yamashiro S, Totsukawa G, Yamakita Y (2003). Citron kinase, a Rho-dependent kinase, induces di-phosphorylation of regulatory light chain of myosin II. Molecular biology of the cell.

[3] Di Cunto F, Calautti E, Hsiao J (1998). Citron rho-interacting kinase, a novel tissue-specific ser/thr kinase encompassing the Rho-Rac-binding protein Citron. The Journal of biological chemistry.

[4] Kosako H, Yoshida T, Matsumura F, Ishizaki T, Narumiya S, Inagaki M (2000). Rho-kinase/ROCK is involved in cytokinesis through the phosphorylation of myosin light chain and not ezrin/radixin/moesin proteins at the cleavage furrow. Oncogene.

[5] Kosako H, Goto H, Yanagida M (1999). Specific accumulation of Rho-associated kinase at the cleavage furrow during cytokinesis: cleavage furrow-specific phosphorylation of intermediate filaments. Oncogene.

[6] Sarkisian MR, Li W, Di Cunto F, D'Mello SR, LoTurco JJ (2002). Citron-kinase, a protein essential to cytokinesis in neuronal progenitors, is deleted in the flathead mutant rat. The Journal of neuroscience: the official journal of the Society for Neuroscience.

[7] Di Cunto F, Imarisio S, Hirsch E (2000). Defective neurogenesis in citron kinase knockout mice by altered cytokinesis and massive apoptosis. Neuron.

[8] Liu H, Di Cunto F, Imarisio S, Reid LM (2003). Citron kinase is a cell cycle-dependent, nuclear protein required for G2/M transition of hepatocytes. The Journal of biological chemistry.

[9] Pardee AB (1989). G1 events and regulation of cell proliferation. Science (New York, N.Y.).

[10] Siegel RL, Miller KD, Jemal A (2017). Cancer Statistics, 2017. CA: a cancer journal for clinicians.

[11] Zigeuner R (2017). Bladder cancer in 2016: News in diagnosis, treatment, and risk group assessment. Nature reviews. Urology.

[12] Wu W, Zhong J, Chen J, Niu P, Ding Y, Han S (2019). Prognostic and Therapeutic Significance of Adhesion-regulating Molecule 1 in Estrogen Receptor-positive Breast Cancer. Clin Breast Cancer. 2019 Sep 3. pii: S1526-8209(19)30654-8. doi: 10.1016/j.clbc.

[13] Irizarry RA, Wang C, Zhou Y, Speed TP (2009). Gene set enrichment analysis made simple, Stat. Methods Med. Res.

[14] Zhang Q, Zhao Z, Ma Y (2014). Combined expression of S100A4 and Annexin A2 predicts disease progression and overall survival in patients with urothelial carcinoma. Urol Oncol.

[15] Madaule P, Furuyashiki T, Eda M, Bito H, Ishizaki T, Narumiya S (2000). Citron, a Rho target that affects contractility during cytokinesis. Microscopy research and technique.

[16] Bassi ZI, Audusseau M, Riparbelli MG, Callaini G, D'Avino PP (2013). Citron kinase controls a molecular network required for midbody formation in cytokinesis. Proceedings of the National Academy of Sciences of the United States of America.

[17] Bassi ZI, Verbrugghe KJ, Capalbo L (2011). Sticky/Citron kinase maintains proper RhoA localization at the cleavage site during cytokinesis. The Journal of cell biology.

[18] D'Avino PP, Savoian MS, Glover DM (2004). Mutations in sticky lead to defective organization of the contractile ring during cytokinesis and are enhanced by Rho and suppressed by Rac. The Journal of cell biology.

[19] Echard A, Hickson GR, Foley E, O'Farrell PH (2004). Terminal cytokinesis events uncovered after an RNAi screen. Current biology: CB.

[20] Gai M, Camera P, Dema A (2011). Citron kinase controls abscission through RhoA and anillin. Molecular biology of the cell.

[21] Gruneberg U, Neef R, Li X (2006). KIF14 and citron kinase act together to promote efficient cytokinesis. The Journal of cell biology.

[22] McKenzie C, Bassi ZI, Debski J (2016). Cross-regulation between Aurora B and Citron kinase controls midbody architecture in cytokinesis. Open biology.

[23] Basit S, Al-Harbi KM, Alhijji SA (2016). CIT, a gene involved in neurogenic cytokinesis, is mutated in human primary microcephaly. Human genetics.

[24] Harding BN, Moccia A, Drunat S (2016). Mutations in Citron Kinase Cause Recessive Microlissencephaly with Multinucleated Neurons. American journal of human genetics.

[25] Shaheen R, Hashem A, Abdel-Salam GM, Al-Fadhli F, Ewida N, Alkuraya FS (2016). Mutations in CIT, encoding citron rho-interacting serine/threonine kinase, cause severe primary microcephaly in humans. Human genetics.

[26] Bianchi FT, Tocco C, Pallavicini G (2017). Citron Kinase Deficiency Leads to Chromosomal Instability and TP53-Sensitive Microcephaly. Cell reports.

[27] D'Avino PP, Capalbo L (2016). Regulation of midbody formation and function by mitotic kinases. Seminars in cell & developmental biology.

[28] Heng HH, Bremer SW, Stevens JB (2013). Chromosomal instability (CIN): what it is and why it is crucial to cancer evolution. Cancer metastasis reviews.

[29] McGranahan N, Burrell RA, Endesfelder D, Novelli MR, Swanton C (2012). Cancer chromosomal instability: therapeutic and diagnostic challenges. EMBO reports.

[30] Fu Y, Huang J, Wang KS, Zhang X, Han ZG (2011). RNA interference targeting CITRON can significantly inhibit the proliferation of hepatocellular carcinoma cells. Molecular biology reports.

[31] D'Avino PP (2017). Citron kinase - renaissance of a neglected mitotic kinase. Journal of cell science.

