# Supplementary material for: Overexpression of Citron Rho-Interacting Serine/Threonine Kinase Associated with Poor Outcome in Bladder Cancer
Source: J Cancer. 2020 Apr 14;11(14):4173–80. doi: 10.7150/jca.43435 (PMC7196275; doi:10.7150/jca.43435)
Supplement: Supplementary file 1 — Supplementary table S1. [file jcav11p4173s1.pdf]

Supplementary Table 1. Summary of download gene expression data sets of Bladder cancer

	GEO				TCGA
Accession No.	GSE13507	GSE31684	E-MTAB-1803	E-MTAB-4321	TCGA
No. of patients	256	93	170	476	131
Assessable cases*	165	93	85	476	129
Date of study	NA	1993-2004	NA	NA	NA
Platforms*	GPL6102	GPL570	NA	NA	NA
Country	South Korea	USA	NA	NA	NA
Gender	Y	Y	Y	Y	Y
Age at diagnosis	66(24-88)	69(41-91)	69(44-89)	69(24-95)	69(34-88)
Grade	Y	Y	Y	Y	Y
Tumor size	NA	NA	NA	Y	NA
TNM stage	Y	NA	NA	NA	Y
Tumor stage	Y	Y	Y	Y	Y
Lymph node	Y	Y	Y	NA	Y
Metas	Y	Y	Y	NA	Y
Tobacco smoking history	NA	Y	NA	NA	Y
Invasiveness	Y	NA	NA	NA	NA
Progression	Y	NA	NA	Y	NA
Recurrence	Y	Y	NA	NA	NA
OS months (Range)	1.03-136.97	0.39-175.50	0-132	NA	0-140.70
Recurrence months(Range)	1.03-136.97	0.39-175.50	NA	NA	2.76-43.97
Progression months(Range)	1.03-136.97	NA	NA	0-74.90	NA

* Platforms: GPL6102:Illumina human-6 v2.0 expression bead chip; GPL570:[HG-U133_Plus_2] Affymetrix Human Genome U133 Plus 2.0 Array
